# Resistance to Erythropoiesis Stimulating Agents in Patients Treated with Online Hemodiafiltration and Ultrapure Low-Flux Hemodialysis: Results from a Randomized Controlled Trial (CONTRAST)

**DOI:** 10.1371/journal.pone.0094434

**Published:** 2014-04-17

**Authors:** Neelke C. van der Weerd, Claire H. Den Hoedt, Peter J. Blankestijn, Michiel L. Bots, Marinus A. van den Dorpel, Renée Lévesque, Albert H. A. Mazairac, Menso J. Nubé, E. Lars Penne, Pieter M. ter Wee, Muriel P. C. Grooteman, CONTRAST Investigators

**Affiliations:** 1 Department of Nephrology, Academic Medical Center, Amsterdam, The Netherlands; 2 Department of Nephrology, VU Medical Center, Amsterdam, The Netherlands; 3 Department of Nephrology, University Medical Center Utrecht, Utrecht, The Netherlands; 4 Department of Internal Medicine, Maasstad Hospital, Rotterdam, The Netherlands; 5 Julius Center for Health Sciences and Primary Care, University Medical Center Utrecht, Utrecht, The Netherlands; 6 Department of Nephrology, Centre Hospitalier de l'Université de Montréal, St-Luc Hospital, Québec, Canada; 7 Institute for Cardiovascular Research VU Medical Center (ICaR-VU), VU Medical Center, Amsterdam, The Netherlands; 8 Department of Internal Medicine, Medical Center Alkmaar, Alkmaar, The Netherlands; Cardiff University, United Kingdom

## Abstract

Resistance to erythropoiesis stimulating agents (ESA) is common in patients undergoing chronic hemodialysis (HD) treatment. ESA responsiveness might be improved by enhanced clearance of uremic toxins of middle molecular weight, as can be obtained by hemodiafiltration (HDF). In this analysis of the randomized controlled CONvective TRAnsport STudy (CONTRAST; NCT00205556), the effect of online HDF on ESA resistance and iron parameters was studied. This was a pre-specified secondary endpoint of the main trial. A 12 months' analysis of 714 patients randomized to either treatment with online post-dilution HDF or continuation of low-flux HD was performed. Both groups were treated with ultrapure dialysis fluids. ESA resistance, measured every three months, was expressed as the ESA index (weight adjusted weekly ESA dose in daily defined doses [DDD]/hematocrit). The mean ESA index during 12 months was not different between patients treated with HDF or HD (mean difference HDF versus HD over time 0.029 DDD/kg/Hct/week [−0.024 to 0.081]; P = 0.29). Mean transferrin saturation ratio and ferritin levels during the study tended to be lower in patients treated with HDF (−2.52% [−4.72 to −0.31]; P = 0.02 and −49 ng/mL [−103 to 4]; P = 0.06 respectively), although there was a trend for those patients to receive slightly more iron supplementation (7.1 mg/week [−0.4 to 14.5]; P = 0.06).

In conclusion, compared to low-flux HD with ultrapure dialysis fluid, treatment with online HDF did not result in a decrease in ESA resistance.

**Trial Registration:**

ClinicalTrials.gov NCT00205556

## Introduction

In chronic hemodialysis (HD) patients, renal anemia is generally treated with erythropoiesis stimulating agents (ESA) and iron supplements. Although treatment with ESA has been shown to improve left ventricular mass index [1] and quality of life [2], ESA treatment has not been associated with a survival benefit, neither in HD patients [3] nor in patients with chronic kidney disease not yet on dialysis [4], [5], [6]. In many HD patients, target hemoglobin levels are not reached due to a varying degree of ESA resistance. A diminished response to ESA has been associated with various factors, including (functional) iron deficiency, an impaired nutritional state and the presence of (micro)inflammation [7], [8]. Furthermore, the microbiological purity of the dialysis fluid [9], the presence of hyperparathyroidism [10] and low dialysis adequacy [11] have been associated with ESA resistance.

Hemodiafiltration (HDF) is an extracorporeal renal replacement therapy that combines diffusive and convective solute removal. In comparison with conventional HD, removal of substances in the middle molecular weight (MMW) range (0.5–40 kilodalton) is enhanced. Several observational studies showed a survival benefit in patients treated with HDF [12], [13], [14], [15], whereas in the randomized controlled CONvective TRAnsport STudy (CONTRAST) study and the Turkish Online Hemodiafiltration Study, survival was similar in patients treated with online HDF and conventional HD [16], [17]. The recently published randomized On-Line Hemodiafiltration Survival Study (ESHOL) did show a survival advantage for patients treated with HDF, however [18].

It has been suggested that adding convective solute removal improves ESA resistance, and hence the degree of anemia, possibly by removing MMW toxins with erythropoiesis inhibiting properties [19], [20], increasing red blood cell life span [21], [22] and/or improving iron utilization [23]. Clinical data on the effect of HDF on ESA resistance, however, are conflicting (table 1).

**Table 1:** Studies on the effect of HDF versus HD on ESA resistance.

	Design	N	Conventional treatment; ultrapure dialysis fluid?^a^	Mean convection volume (L/session)	Outcome
Bonforte 2002 [37]	Observational	32	Low-flux HD; no	19.5; post-dilution	↓ ESA resistance
Lin 2002 [23]	Cross-over	92	High-flux HD; yes	21; post-dilution	↓ ESA resistance
Vaslaki 2006 [36]	Randomized cross-over	129	Low-flux HD; no	20.2; post-dilution	↓ ESA resistance
Schiffl 2007 [35]	Randomized cross-over	76	Low-flux HD; no	20.3; post-dilution	↓ ESA resistance
Turkish online HDF study 2013 [17]	RCT	782	High-flux HD; no	21.0; post-dilution	↓ ESA resistance
Vilar 2009 [15]	Observational	858 (232 HDF)	High-flux HD; yes	Not available	No difference
Oates 2011 [38]	Observational	34 HDF, 44 HD	High-flux HD; yes	>16; post-dilution	No difference
Ward 2000 [39]	RCT	44	High-flux HD; yes	18.0; post-dilution	No difference
Wizemann 2000 [40]	RCT	44	Low-flux HD; yes	60; mid-dilution	No difference
Locatelli 2012 [41]	RCT	146	Low-flux HD; yes	HF: 60.4, HDF: 39.9; pre-dilution	No difference
ESHOL 2013 [18]	RCT	906	High-flux HD; yes	23.4; post-dilution	No difference
CONTRAST 2013	RCT	714	Low-flux HD; yes	20.3; post-dilution	No difference

a Ultrapure dialysis fluid is defined as <0.1 colony forming units per ml and <0.03 endotoxin units per ml [28].

Anemia management and ESA resistance were predefined secondary endpoints of the CONTRAST study [16], [24]. In the present analysis of this randomized controlled trial (RCT), the effect of online post-dilution HDF and ultrapure low-flux HD on hemoglobin levels, ESA resistance and iron parameters was evaluated over a 12 month period.

## Materials and Methods

### Patients and study design

In the CONTRAST study (clinicaltrials.gov identifier NCT00205556; first registration on September 2005), 714 chronic HD patients were randomized either to switch to online HDF or to continue low-flux HD. The protocol for this trial and supporting CONSORT checklist are available as supporting information; see Checklist S1 and Protocol S1. The study was conducted in accordance with the Declaration of Helsinki and was approved by a central medical ethics committee and by all local medical ethics review boards. Written informed consent was obtained from all patients prior to enrolment. Central approval was obtained by the Medical Ethical Committee of the VU University Medical Center. Local approval was obtained from the Medical Ethical Committees of all participating centers, which are listed in the acknowledgements. The enrolment period started in June 2004 and ended on January 1^st^ 2010. The study stopped on January 1^st^ 2011. For the present analysis, all available data on anemia management and ESA resistance until 12 months of follow-up were evaluated. Anemia management and ESA resistance was a pre-specified secondary endpoint of the study and as such registered on clinicaltrials.gov. The trial registration date of the study was slightly after enrolment started due to logistic reasons. The authors confirm that all related trials for this treatment intervention are registered.

The rationale and the design of the CONTRAST study have been described before [24]. Primary endpoint of the study is all cause mortality [16]. Patients were recruited from 26 dialysis centers in The Netherlands, 2 centers in Canada and 1 center in Norway and randomized centrally into a 1∶1 ratio for treatment with online HDF or continuation of low-flux HD, stratified per participating center. Patients were eligible for inclusion if they were treated two or three times per week with HD for at least two months. Exclusion criteria were age below 18 years, treatment with hemo(dia)filtration or high-flux HD in six months prior to randomization, a life expectancy less than three months due to non-renal disease, participation in another clinical intervention trial evaluating cardiovascular outcomes and severe incompliance regarding frequency and/or duration of dialysis treatment.

### Treatment protocol

Upon randomization, all patients were stable with a minimum dialysis spKt/V_urea_ of 1.2 per treatment. Treatment times were fixed at baseline and could only be increased during follow-up when the spKt/V dropped below 1.2. Online HDF was performed in post-dilution mode with a target dose for convection flow of 100 mL/min and high-flux synthetic dialyzers were used (UF coefficient 55–85 ml/mmHg/h: FX80, FX100 and Optiflux F200NR [Fresenius Medical Care, Bad Homburg, Germany] and Polyflux170H and Polyflux210H [Gambro Corporation AB, Lund, Sweden]). Conventional HD was performed with low-flux synthetic dialyzers (UF coefficient 10–18 ml/mmHg/h: F6HPS, F8HPS and Optiflux18NR [Fresenius] and Polyflux14L and Polyflux17L [Gambro]). Routine patient care was performed according to the opinion of the attending nephrologist and based on the Quality of Care Guidelines of the Dutch Federation of Nephrology. The Dutch Guideline on Anemia Management is derived from the European Best Practice Guidelines [25] and the KDOQI guidelines [26], [27].

ESA and iron supplements were administered via the venous blood line at the end of a dialysis session. Both HDF and HD patients were treated with ultrapure dialysis fluids, containing less than 0.1 colony forming units per mL and less than 0.03 endotoxin units per mL [28], [29]. The quality of the dialysis solutions was regularly monitored as part of the Quality of Care Guideline on water quality of the Dutch Federation of Nephrology [29].

### Data collection

At baseline, data on demography, cause of renal failure, history of cardiovascular disease, diabetes mellitus, type of vascular access, dialysis vintage and treatment parameters were collected. Subsequent study visits were performed at three month intervals. During these visits, information on ESA and iron doses at the time of the visit date was collected.

ESA was prescribed as epoetin α or β (IU) or darbepoetin α (µg). To compare the different types of ESA, weekly prescribed dosages were converted to daily defined doses (DDD), using conversion factors as provided by the World Health Organization (WHO) Drug Classification (http://www.whocc.no/atcddd). DDD represents the assumed average maintenance dose per day for a drug used for its main indication in adults. For darbepoetin α (ATC code B03XA02) DDD is 4.5 µg and for epoetin α and β (ATC code B03XA01) DDD is 1000 IU.

ESA resistance was expressed as an ESA index: the weekly weight adjusted ESA dose (DDD) divided by the haematocrit (Hct) [30]. Iron supplements were prescribed as iron sucrose or iron dextran (in mg/week), both with an equal amount of elemental iron per mg.

At each study visit, with a window of two weeks around the exact visit date, blood samples were drawn prior to dialysis for routine laboratory assessments. All laboratory samples were analysed in the local hospitals by standard laboratory techniques. Ferritin levels (µg/L) and the transferrin saturation ratio (TSAT) were used as indices of iron stores. TSAT values were either reported directly or calculated as serum iron divided by the serum total-iron-binding capacity (TIBC), which was considered to represent serum transferrin level [31].

Serum albumin was measured with either the bromcresol green or bromcresol purple method and bromcresol purple concentrations were converted to bromcresol green [32]. The second generation Daugirdas formula was used to calculate spKt/V_urea_ [33]. In patients treated with HDF, the average achieved convection volume per session was calculated as the sum of the intradialytic weight loss and the infusion volume (substitution volume). The HDF treatment was quantified by the convection volume and pre-dialysis levels of β2-microglobulin (β2M; mg/L) over time [34].

### Statistical analysis

All analyses were conducted according to an intention-to-treat principle. Patient data were censored at the date of death or date of the last study visit if the patient stopped with the study. All variables were reported as proportions or means ± standard deviation (SD) or 95% confidence interval (CI), or medians with 25^th^–75^th^ percentiles when appropriate.

Laboratory parameters, ESA dose and -index and iron supplementation over time were evaluated with linear mixed-effects models, as repeated measurements over time were assumed to be related within participants. All available measurements until 12 months of follow-up were entered into the models. For each individual, a mean level of the laboratory parameters during the follow-up was estimated. Subsequently, the difference in the mean levels between the treatment groups was calculated, adjusted for the baseline levels.

To evaluate a possible dose-response relation in HDF patients, the effect of treatment on change in ESA index was explored in different tertiles of convection volume during 12 months of follow-up. Low-flux HD was regarded as the reference category. The choice for tertiles was based on the reasonable sample size in each of the tertiles.

To evaluate whether the treatment effect on mean ESA index was modified by the stratum of ESA resistance at baseline, the possibility of effect modification was explored by adding an interaction term (ESA index×treatment modality) to the linear mixed-effects model.

Since a total of nine parameters were evaluated, we applied a Bonferroni adjustment for multiple testing and considered a two sided p-value<0.006 as statistically significant. Statistical analyses were performed with SPSS software (version 20, 2011, IBM SPSS Statistics) and R (version 2.9.2; 2009 The R Foundation for Statistical Computing).

## Results

### Patient characteristics and clinical parameters at baseline

**Figure 1:**
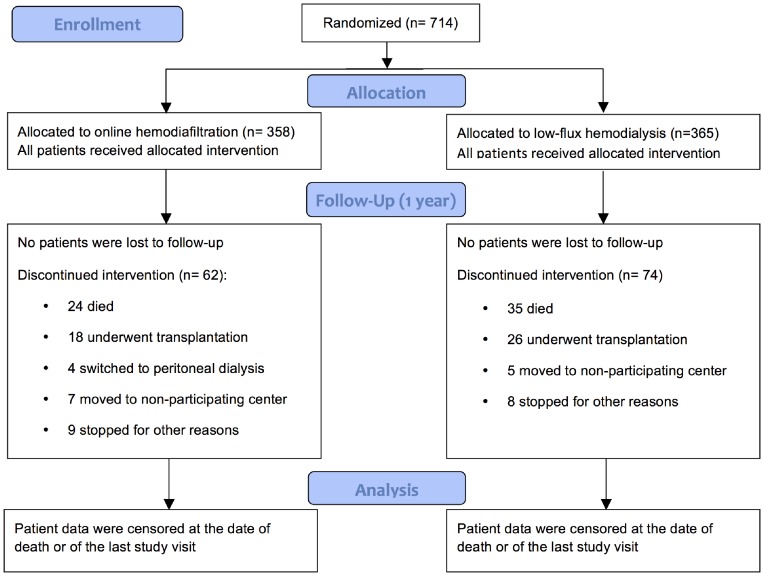
CONSORT flow chart of patient inclusion.

In total, 714 patients were included in the study, 358 were allocated to HDF and 356 to HD. The two groups were well balanced with respect to baseline characteristics (table 2). During 12 months of follow-up, 62 patients in the HDF group and 74 in the HD group were censored because they died, received a kidney transplant, switched to peritoneal dialysis, moved to another center or for other reasons (figure 1).

**Table 2:** Baseline characteristics^a^.

	Online HDF (n = 358)	Low-flux HD (n = 356)
Gender (no. [%] male)	214 (60)	231 (65)
Age (years)	64.1±14.0	64.0±13.4
Caucasian race (no. [%])	304 (85)	296 (83)
Dialysis vintage (years)	1.8 (1.0–3.7)	2.1 (1.0–4.0)
Cause of ESRD (no. [])		
- vascular	104 (29)	96 (27)
- diabetes mellitus	76 (21)	60 (17)
- glomerulonephritis	36 (10)	53 (15)
- interstitial nephropathy	35 (10)	31 (9)
- multisystem disease	15 (4)	11 (3)
- cystic disease	27 (7)	26 (7)
- other/unknown	65 (18)	79 (22)
Diabetes mellitus (no. [%])	92 (26)	78 (22)
History of cardiovascular disease (no. [%])^b^	141 (42)	162 (46)
Body weight after dialysis (kg)^c^	71.6±15.0	73.3±13.6
BMI after dialysis (kg/m^2^)	25.2±5.0	25.6±4.6
Residual kidney function (no. [%])^d^	186 (52)	190 (53)
eGFR (ml/min/1.73 m^2^)^e^	0.32 (0–3.30)	0.30 (0–3.35)
Treatment frequency (no. [%])		
- 2×/week	26 (7)	18 (5)
- 3×/week	332 (93)	338 (95)
Duration of a dialysis session (min)	226±26	227±22
Bloodflow (mL/min)	302±39	299±41
Dialysis access (no. [%])		
- AV fistula	279 (78)	288 (81)
- graft	57 (16)	43 (12)
- central catheter	22 (5)	25 (7)
spKt/V_urea_	1.42±0.25	1.38±0.20
Hemoglobin (g/dL)	11.9±1.3	11.8±1.1
Hematocrit	0.36±0.04	0.36±0.04
Ferritin (ng/mL)		
- Median (interquartile range)	314 (191–567)	367 (196–606)
- Mean ± SD	423±347	442±322
TSAT (%)	23.9±11.1	24.4±11.7
Albumin (g/dL)	3.66±0.45	3.70±0.46
Parathyroid hormone (pg/ml)	193.7 (94.0–322.9)	194.7 (104.5–355.2)
β2-microglobulin (mg/L)	30.7±14.2	32.3±13.6
ESA treatment (no. [%])	311 (87)	320 (90)
Type of ESA (no [%])		
- Darbepoetin α	226 (73)	231 (72)
- Epoetin β	64 (20)	71 (22)
- Epoetin α	21 (7)	18 (6)
ESA dose (DDD/week)		
- Median (interquartile range)	8.0 (4.0–13.3)	7.6 (4.4–13.3)
- Mean ± SD	9.7±9.0	9.8±8.1
ESA index (DDD/kg/Hct/week)		
- Median (interquartile range)	0.31 (0.14–0.59)	0.30 (0.15–0.55)
- Mean ± SD	0.40±0.39	0.40±0.37
Iron replacement (no. [%])	236 (66)	231 (65)
Type of iron replacement (no. [%])		
- Ironsucrose	215 (91)	213 (92)
- Irondextran	21 (9)	18 (8)
Iron dose (mg/week)		
- Median (interquartile range)	23.3 (0–100)	23.3 (0–100)
- Mean ± SD	48±51	43±42

a Values represent mean ± SD, median (interquartile range) or proportion (%).

b History of cardiovascular disease was defined as history of angina pectoris or myocardial infarction and/or a previous coronary bypass graft and/or percutaneous coronary intervention and/or stroke or transient ischemic attack and/or peripheral vascular disease.

c Weight after dialysis (dry weight) defined as the mean of three consecutive values.

d Defined as >100 mL per day.

e eGFR (estimated glomerular filtration rate) calculated as mean of creatinine and urea clearance in 24 h urine collection adjusted for body surface area [48].

Conversion factors for units: hemoglobin in g/dL to mmol/L, ×0.62; albumin in g/dL to g/L, ×10; parathyroid hormone pg/ml to pmol/l ×0.11; no conversion necessary for ferritin in ng/mL and µg/L.

### Clinical parameters during follow-up

For patients treated with HDF, the mean (± SD) achieved convection volume during one year of follow-up was 20.3±4.8 liters per session. During the study, the mean dialysis single pool Kt/V urea (spKt/V_urea_) was higher in in the HDF group (mean [95% CI] difference HDF vs. HD 0.17 [0.07 to 0.27; P<0.001]). The mean β2M level during follow-up was significantly lower in the HDF group (mean difference HDF vs. HD −7.8 mg/L [−10.5 to −4.9; P<0.001]). Furthermore, albumin levels were equal in both groups during follow-up (mean difference HDF vs. HD −0.02 g/dL [−0.06 to 0.02; P = 0.35]).

#### Hemoglobin and ESA resistance

**Figure 2:**
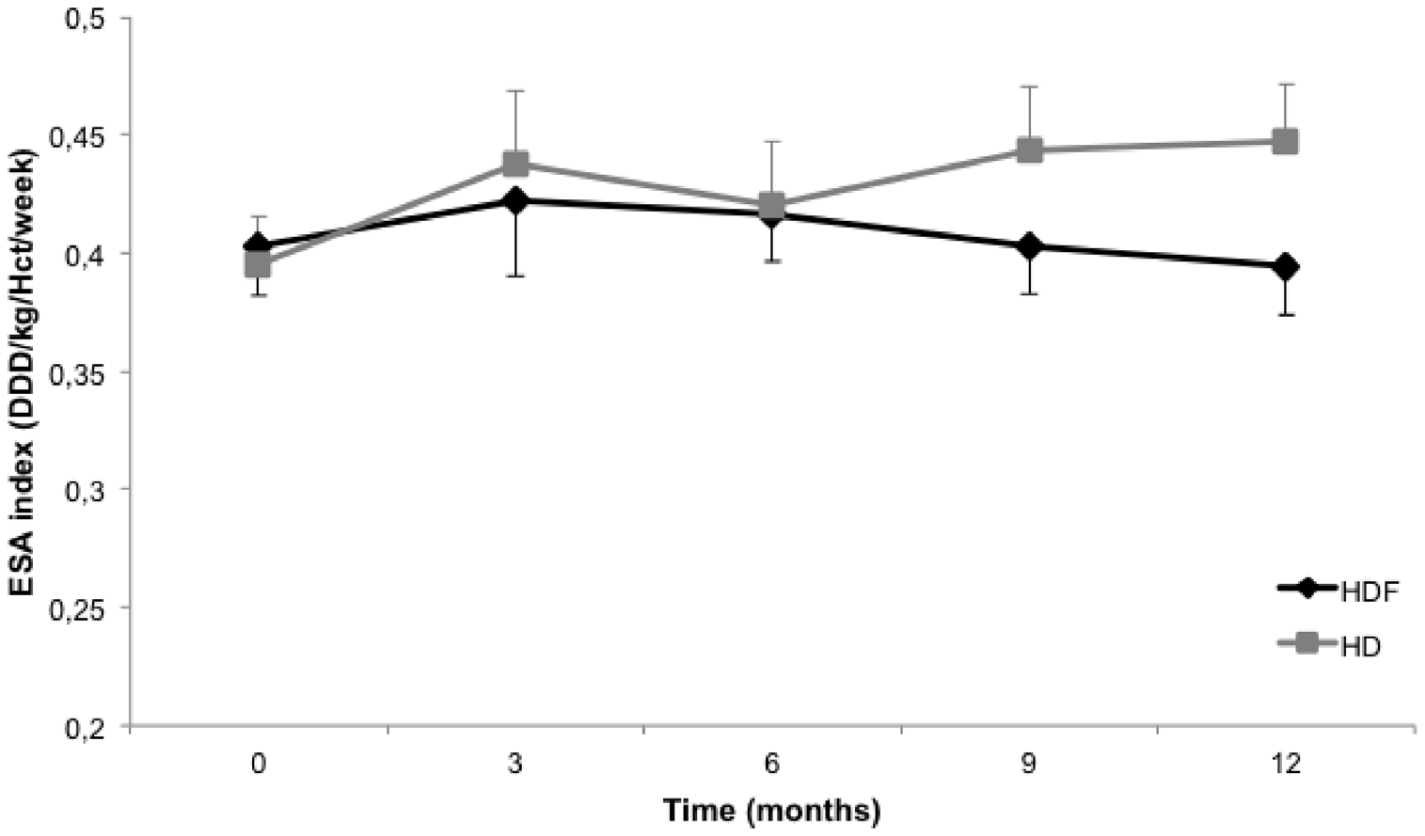
Change in ESA index for HDF and HD. Crude means (SE) at time of visit are depicted. P-value for mean difference in ESA index over time (HDF vs. HD) = 0.29 (based on a linear mixed-effects model including all 3122 measurements during 12 months of follow-up).

Anemia parameters during follow-up are listed in table 3. Hemoglobin levels were not different between both groups. Furthermore, there was no difference in mean ESA index and ESA dose between the treatment groups during follow-up (table 3, figure 2).

**Table 3:** Mean of the parameters of anemia management during follow-up period, by treatment assignment.

	Online HDF^a^	Low-flux HD^a^	Difference HDF vs HD^b^	
	Mean value (95% CI)	Mean value (95% CI)	Mean value (95% CI)	P-value
Hemoglobin (g/dL)	11.9 (11.8 to 12.0)	11.7 (11.6 to 11.8)	0.1 (−0.2 to 0.3)	0.63
Hematocrit	0.36 (0.35 to 0.36)	0.35 (0.35 to 0.36)	0.003 (−0.004 to 0.010)	0.48
Ferritin (ng/mL)	441 (409 to 473)	472 (440 to 504)	−49 (−103 to 4)	0.06
TSAT (%)	23.1 (22.1 to 24.1)	24.3 (23.4 to 25.3)	−2.5 (−4.7 to −0.3)	0.02
ESA index (DDD/kg/Hct/week)	0.41 (0.38 to 0.45)	0.43 (0.39 to 0.47)	0.03 (−0.02 to 0.08)	0.29
ESA dose (DDD/week)	10.1 (9.3 to 10.9)	10.4 (9.5 to 11.2)	0.9 (−0.2 to 2.0)	0.12
Iron dose (mg/week)	45 (41 to 49)	35 (32 to 38)	7.1 (−0.4 to 14.5)	0.06

a Crude mean (95% CI) of values at 3, 6, 9 and 12 months.

b Difference in mean values during the follow-up period between groups as a result from a linear mixed-effects model adjusted for the value at baseline, including all measurements at 3, 6, 9 and 12 months.

To explore a possible effect of the HDF dose, expressed as the average achieved convection volume during follow-up, the change in ESA index was analyzed separately within tertiles of convection volume. Within each tertile of achieved convection volume, the ESA index did not change and was not different compared to HD (data not shown).

#### Iron parameters and iron therapy

Ferritin levels at baseline were not different between both groups (HDF: (median [interquartile range]: 314 ng/mL [191–567]; HD 367 ng/mL [196–606]; P = 0.16). During one year of follow-up, mean ferritin and TSAT levels tended to be marginally lower in the HDF group as compared to HD (mean difference HDF vs. HD −49 ng/mL [−103 to 4; P = 0.06] and −2.5% [−4.7 to −0.3; P = 0.02] respectively; table 3).

The mean dose of iron supplementation during follow-up tended to be slightly higher in the HDF group as compared to HD (mean difference HDF vs. HD 7.1 mg/week [−0.4 to 14.5; P = 0.06]).

#### Sub-analysis: ESA resistance over time in different groups of ESA index at baseline

The effect of HDF versus HD on mean ESA index over time was not modified by the ESA index at baseline (P-value for the interaction term = 0.15).

## Discussion

The present secondary analysis of a RCT showed that ESA resistance over a 12 months' period, as measured by the mean ESA index over time, was not different between patients treated with online HDF or HD. In the HDF group, iron parameters (ferritin and TSAT) tended to be slightly lower during follow up whereas there was a trend towards more intravenous iron supplementation in this group.

As mentioned before, studies on the effect of HDF on ESA resistance have been conflicting (table 1). Our overall results are in contrast with results of two cross-over studies [35], [36], two observational studies [23], [37] and one RCT [17] in which ESA dose and/or ESA resistance decreased and/or hemoglobin levels increased in patients treated with online HDF. On the contrary, in two non-randomized studies [15], [38] and two small [39], [40] and two large RCTs [18], [41], no difference between treatment with HDF or HD in either ESA dose or hemoglobin levels was shown either. Remarkably, in virtually all studies in which a beneficial effect of HDF on ESA resistance was shown, the dialysis fluid applied in the conventional HD group was not ultrapure according to the generally accepted definition (<0.1 colony forming units per ml and <0.03 endotoxin units per ml) [28]. In contrast, in the studies that did not show a difference, ultrapure dialysis fluid was used for both treatment with HD and HDF (table 1). A recent meta-analysis has shown that the use of ultrapure dialysis fluid resulted in increased hemoglobin levels and diminished ESA requirements [9]. Furthermore, in a recently published RCT in 704 patients, it was shown that ultrapure dialysis fluid as compared to standard dialysis fluid resulted in reduced ESA doses and ESA resistance despite equal hemoglobin and ferritin levels [42]. Therefore, we hypothesized that the use of ultrapure dialysis fluid may be of predominant importance with respect to erythropoiesis and ESA responsiveness as compared to increasing MMW clearance by adding convection [43], [44], [45].

Another possible explanation for the absent effect of HDF on ESA resistance may be that with HDF, besides removal of ESA or erythropoiesis inhibiting toxins, erythropoiesis stimulating substances are removed as well. However, we have no data to support this hypothesis.

We postulated that the amount of convection volume, being a key quantifier for HDF treatment according to a recent position statement by the HDF working group (EUDIAL) of the European Renal Association-European Dialysis and Transplant Association [34], might affect ESA responsiveness. In an on-treatment analysis of both CONTRAST and the Turkish HDF study, a survival benefit for HDF patients treated with the highest convection volumes was observed [16], [17]. In the ESHOL study, in which an overall survival benefit for HDF was shown, convection volumes were higher (23.4 L per session versus 19.8 L and 21.04 in CONTRAST and the Turkish study, respectively) [18]. Considering convection volume and ESA resistance, it was shown in a study in 37 chronic dialysis patients that a switch from treatment with low-volume HDF (mean substitution volume 4.0 L/session) to high-volume online HDF (mean substitution volume 22.5 L/session) resulted in increased hemoglobin levels and decreased ESA doses. In our study, however, we could not identify such a dose-response effect with respect to ESA resistance.

Concerning iron therapy, it was previously reported that ferritin and TSAT levels decreased in HDF patients despite a lower ESA dose and a higher haematocrit, suggesting improved iron utilization [23]. As mentioned, in our study, HDF patients tended to receive slightly more intravenous iron than HD patients which might be partially explained by relatively low ferritin levels in HDF patients at baseline, suggesting that overall, patients randomized to HDF were more iron deplete at the time of enrolment. This relative iron deplete state seemed to remain present during the study. Other potential explanations for the relative iron deplete state in the HDF group despite slightly more iron supplementation might be loss of ascorbic acid (vitamin C) [46], since this vitamin enhances iron availability [47], [48], and blood loss due to clotting in the extracorporeal circuit as a result of increased pro-coagulatory activity during HDF [49].

We restricted the analysis to one year of follow-up since previous studies already showed an effect of HDF on ESA resistance after 3 to 6 months [36], [37]. A limitation of our study is the conversion of ESA dose to DDD, which may be an oversimplification, as a non-linear conversion factor, dependent upon the actual ESA dose, has been described [50]. An additional drawback is the lack of a specific treatment protocol for ESA and iron administration. However, as the treatment allocation was stratified per participating center, the effect on the study outcome will probably be limited. Moreover, our study reflects usual care in the Netherlands. In our study, HDF was compared with low-flux HD although at present, the majority of patients is treated with high-flux dialyzers as recommended in current guidelines [51]. Since we observed no difference between HDF and low-flux HD concerning ESA resistance, it is highly unlikely that there would be a difference when HDF and high-flux HD (which can be considered as a form of “low-volume HDF”) were compared. Furthermore, in the ESHOL study, no effect of HDF versus high-flux HD on ESA resistance was observed either [18]. A final limitation is the handling of missing data due to drop out. These data might be missing in a non-randomly fashion.

The strength of our study is the large sample size and its randomized design with an accurate prospective data collection. Furthermore, all available measurements during follow-up (at 3, 6, 9 and 12 months) were included in the analysis.

In conclusion, this multicenter randomized controlled study showed that ESA resistance was not different between patients treated with online HDF or ultrapure low-flux HD during 12 months of follow-up. These data indicate that when considering ESA resistance, online HDF has no benefit over low-flux ultrapure HD. Whether other factors are involved, such as the quality of the dialysis fluid, remains to be determined.

## Supporting Information

Checklist S1 **CONSORT Checklist.**
(DOC)

Protocol S1 **Trial Protocol.**
(DOC)
